# Assessment of the Quality of Life and Communication Needs of Deaf Ecuadorians

**DOI:** 10.3390/ejihpe16060082

**Published:** 2026-06-13

**Authors:** Emily Jo Noschese, Alina Engelman, Leah R. Oakes, Lorne Farovitch

**Affiliations:** 1 College of Education, Wayne State University, 5425 Gullen Mall, Detroit, MI 48202, USA; 2 Department of Public Health, California State University, East Bay, 25800 Carlos Bee Blvd, Hayward, CA 94542, USA; alina.engelman@csueastbay.edu; 3 Department of Theory, Practice & Teacher Education, University of Tennessee, 1126 Volunteer Blvd, Knoxville, TN 37916, USA; lroakes@vols.utk.edu; 4 Global Deaf Research Institute, 2028 E Ben White Blvd #240-7215, Austin, TX 78741, USA; farovitch@globaldeafresearch.org

**Keywords:** deaf, hard-of-hearing, Ecuador, quality of life, access, disability, communication, employment, education, healthcare access, interviews, structural vulnerability, adverse childhood communication experiences

## Abstract

Deaf people experience significant barriers to education, healthcare, employment, and information access, resulting in inequities across a myriad of contexts. To better understand these disparities, our all-deaf research team conducted semi-structured interviews with deaf and hearing (parents, caregivers, and educators) adults across Ecuador, exploring how structural, institutional, and social factors influence daily life and well-being. Participants (*n* = 36) described systemic exclusion from education and employment, limited access to interpreters and assistive technologies, and constrained autonomy due to insufficient family support and institutional resources. These barriers compound health risks by restricting access to care, information, and social participation. Participants’ narratives highlighted how political and economic instability, institutional neglect, and discrimination create structural vulnerabilities that extend beyond individual-level factors. Findings underscore the importance of public health interventions that address structural and communicative inequities, including inclusive education, accessible health services, and community-based support, to improve health equity and quality of life for deaf populations in Ecuador.

## 1. Introduction

Quality of life reflects the conditions that enable autonomy, well-being, and active participation within communities (Kaplan & Hays, 2022). It is widely understood as a multidimensional construct encompassing physical health, psychological well-being, social relationships, and environmental conditions. In disability research, quality of life is shaped not only by individual health status but by the degree to which social and institutional environments enable or constrain participation and self-determination (Degener, 2016; Schalock et al., 2002). For deaf individuals specifically, quality of life is further mediated by communication access; the extent to which environments, institutions, and relationships accommodate deaf ways of communicating shapes opportunities for participation, autonomy, and well-being across the lifespan (Kuenburg et al., 2015; Kushalnagar et al., 2020).

It is also shaped by access to essential services and resources, including stable housing, transportation, education, employment, public services, and healthcare. For deaf and disabled people worldwide, these forms of access are not determined at the individual level but are structured by the stability of broader political, economic, and institutional systems (Degener, 2016; Engelman et al., 2025a; Izquierdo Martínez et al., 2024; Jaiyeola & Adeyemo, 2018; Kuper et al., 2024). When access to services is limited or unreliable, deaf and disabled individuals’ ability to participate fully in social, economic, and public life is likewise constrained. Deaf people are among those who have been impacted by these conditions, as everyday participation in education, employment, healthcare, and public services frequently depends on the availability of effective communication access (Engelman et al., 2025a; Izquierdo Martínez et al., 2024; Jaiyeola & Adeyemo, 2018). As a result, broader structural conditions play a significant role in shaping deaf people’s quality of life; our research team aimed to gain deeper insight into these experiences among PWD and deaf people in Ecuador, resulting in the study presented here.

### 1.1. Deafness and Structural Inequalities

Language deprivation and communication neglect in the family, best understood as adverse childhood communication experiences, can impact major health outcomes across the lifespan (Kushalnagar et al., 2020). For deaf people, structural barriers are often compounded by the added dimensions of communication access, knowledge gaps, and social stigma, as the public lacks understanding of both deafness itself and evidence-based practices for effective communication. Gaps in awareness and education about deafness among the general population, in particular, shape how institutions respond to deaf individuals, often reinforcing communication and access barriers (Andrade Pereira & De Carvalho Fortes, 2010; W. C. Hall, 2017; Plaza-Pust & Morales-López, 2008). Deafness can be understood not as an individual characteristic, but as a socially established experience shaped by structural and institutional arrangements (McIlroy & Storbeck, 2011). Systems of access, participation, and support are frequently designed without deaf people in mind.

Deaf people have historically been underserved, particularly those with intersecting marginalized identities (Crenshaw, 1991), within broader structural inequalities. Social and institutional systems have been largely designed around hearing communication norms (Barnett et al., 2011; Hauser et al., 2010; Renel, 2019). The limited recognition of sign languages, combined with inconsistent access to interpretation, assistive communication technologies and services, and information, has often shaped unequal access to essential services (Barnett et al., 2011; Iezzoni et al., 2004; Meulder, 2015; Sweet et al., 2019). In many institutional contexts, the absence of formal recognition translates into improvised or inadequate communication accommodations, placing responsibility on individuals rather than systems to advance despite barriers (Calgaro et al., 2021; Harmer, 1999; Steinberg et al., 2006). As a result, deaf people may encounter barriers to classroom instruction, workplace participation, healthcare decision-making, and engagement with public institutions, contributing to persistent inequities in opportunity, which in turn affect quality of life. Limited access to timely and accessible information, including during emergencies, further constrains long-term resilience and safety, often leaving deaf individuals among the last to receive critical updates and guidance (Calgaro et al., 2021; Engelman et al., 2025b).

### 1.2. Ecuadorian Context

Within Ecuador, political and economic conditions, as well as infrastructure, shape how people experience access to essential services. Ecuador is an upper-middle-income country with ongoing challenges related to income inequality and multidimensional poverty, particularly in rural areas where 43.2% of the population face barriers to education, basic services, and economic opportunities (World Bank, 2024a). Multidimensional poverty^1^ affects a large portion of Ecuador’s population, with residents in rural areas experiencing higher levels of deprivation than those in urban settings (United Nations Development Programme, 2023; World Bank, 2024a).

Economic conditions in Ecuador have been volatile, influenced by dependence on extractive exports and external demand, and constrained public spending that shapes infrastructure and the delivery of social services (Commonwealth & Development Office et al., 2023; World Bank, 2024b). Social vulnerability remains spatially uneven, with rural communities often facing inadequate housing, limited basic services, and weaker institutional support compared to urban areas (Torres-Díaz et al., 2025). Ecuador has experienced security challenges, economic uncertainty, and contested policy reforms that have affected governance and trust in public institutions (Bertelsmann Stiftung, 2024; Binev, 2024; Le Monde, 2025). Public spending on services such as healthcare has been uneven, and reductions in health sector funding have coincided with health infrastructure and management challenges (Bertelsmann Stiftung, 2024).

### 1.3. Deaf Ecuadorians

For deaf individuals in Ecuador, these broader conditions intersect with persistent barriers to communication and information access, which shape everyday participation and quality of life. Research has shown that communication barriers affect access to essential services and public life, positioning deaf people at a structural disadvantage relative to the general population (30–32). Limited recognition of sign languages, inconsistent access to interpretation, assistive communication technologies and services, and restricted access to accessible information, including critical public service and emergency communication, often leave deaf individuals reliant on informal networks, further reinforcing patterns of exclusion and inequality.

Empirical knowledge about deaf people in Ecuador remains limited. Sociolinguistic surveys have documented sign language use and community characteristics, providing foundational context for the Ecuadorian deaf community (Eberle et al., 2012). Qualitative research has largely focused on family perspectives, highlighting parents’ and grandparents’ concerns regarding education, access to services, and social inclusion (Huiracocha et al., 2015; Huiracocha-Tutiven et al., 2017). More recent survey-based research has documented communication barriers and variable satisfaction in healthcare settings, but this work does not capture participants’ own interpretive accounts of barriers across different domains of daily life (Izquierdo-Condoy et al., 2024).

A national deaf needs assessment in 2026 reinforces these gaps, showing that 88% of deaf Ecuadorians reported being unable to obtain the accommodations they needed in primary school, with educational attainment largely limited to primary (32%) or secondary education (27%) and only 14% reporting access to higher education. Economic exclusion is also evident, with 41% of respondents unemployed or working without pay and only 7% reporting that they were able to meet their financial needs (Global Deaf Research Institute [GDRI], 2026). Transportation further compounds these challenges, as 86% expressed dissatisfaction with transportation, and 48% reported it was unaffordable (Global Deaf Research Institute [GDRI], 2026). Taken together, these studies suggest that while the Ecuadorian deaf community has been studied, research gaps remain in understanding how deaf individuals themselves experience structural inequalities and perceive their own quality of life.

Deaf associations and community organizations in Ecuador provide support, advocacy, and opportunities for community engagement, including sign language education, awareness campaigns, and guidance in accessing information and services. For example, Robles-Bykbaev et al. (2019) describe a bespoke social network created for deaf women in Ecuador to access information on sexual and reproductive health, emphasizing how community-driven initiatives can partially mitigate gaps in formal communication and service provision. However, they also stress the persistence of structural inequities, such as individuals who must rely on community-based solutions in the absence of consistent institutional support. However, reliance on community organizations also reflects systemic gaps, as responsibility for communication access and inclusion is often shifted away from public institutions and onto deaf communities or deaf-led nonprofit organizations themselves (Huiracocha-Tutiven et al., 2017; Meulder, 2015; Robles-Bykbaev et al., 2019). Yet, these organizations’ limited capacity cannot fully compensate for inconsistent institutional policies or limited legal enforcement. 

### 1.4. Structural Vulnerability Framework

This study is informed by the Structural Vulnerability Framework (Quesada et al., 2011), which emphasizes how political, economic, and legal structures produce patterned disadvantages for marginalized populations. The framework argues that individuals’ vulnerability to harm is not randomly distributed but is systematically produced by their position within interlocking political, economic, and social structures. Structural position, shaped by factors such as legal status, race, class, language, and disability, determines differential exposure to risk, limited access to resources, and reduced capacity to resist or recover from harm. Importantly, the framework locates the source of vulnerability in these structures themselves rather than in individual behaviors or characteristics, making it particularly well-suited for analyzing the experiences of marginalized populations whose disadvantages are produced and maintained by institutional arrangements.

This structural positioning has direct consequences for quality of life. When political, economic, and institutional conditions systematically restrict access to education, healthcare, employment, and communication, the conditions necessary for autonomy, well-being, and social participation are undermined at the structural level rather than the individual level (Degener, 2016; Kaplan & Hays, 2022). For deaf individuals, communication access functions as a key structural determinant of quality of life; its presence or absence shapes whether deaf people can participate meaningfully in the institutions and relationships that enable well-being. Applied to deaf communities in Ecuador, this framework treats each of the five themes identified in this study, as described in the Results section, as a distinct axis through which structural positioning shapes quality of life.

## 2. Methods

This study was conducted to document the lived experiences, needs, and quality of life of deaf communities globally. To better understand the experiences of deaf communities in Ecuador, we designed this qualitative study to center participants’ perspectives on accessibility, communication, available services and resources, and conditions of daily life.

All members of the research team are deaf and bring interdisciplinary training in deaf education, linguistics, public health, and biomedical sciences, along with longstanding experience working alongside and within deaf communities through research, advocacy, and education. Our positionalities (Creswell & Poth, 2018) as deaf researchers, shaped by shared and divergent experiences across disability, language use, and other layered identities, informed the design, conduct, and interpretation of this study, as well as the development of rapport and trust during data collection.

Approaching this work from a community-engaged and rights-based framework (Israel, 2013), we recognized that reflexive engagement with our positionalities was essential, even where meaningful convergence existed between participants and researchers. This reflexivity shaped the study design, recruitment strategies, and interview process, which collectively prioritized cultural respect, linguistic access, and participant trust. To that end, we engaged closely and frequently with local deaf communities and organizations prior to data collection, meeting with community members and stakeholders to ensure that study questions and procedures reflected community priorities and realities. This study was reviewed and approved by the WCG Institutional Review Board (IRB) (Protocol #20243931).

### 2.1. Recruitment Procedures

Participants were recruited through community outreach, including collaboration with deaf community networks and word-of-mouth referrals, with snowball sampling (Patton, 2015) employed once data collection began. The rapport and trust built through prior community engagement served as a foundation for recruitment, facilitating access and encouraging participation. Recruitment materials were made available in both written Spanish and Ecuadorian Sign Language (LSEC).

Prior to each interview, participants were informed of the study purpose, procedures, risks, and benefits in both written Spanish and LSEC. Informed consent was obtained using a standardized research consent form, in accordance with established standards for research with deaf communities (Kosa et al., 2023). Participation was voluntary, and participants were informed of their right to decline any question or withdraw at any time without penalty.

### 2.2. Participants

Participants were drawn from multiple regions across Ecuador, including both urban and rural areas, to capture a diversity of experiences related to geography, access to services, and community infrastructure. Inclusion criteria required participants to be 18 years of age or older, residing in Ecuador, and either deaf or hard-of-hearing themselves, or a hearing relative, caregiver, or educator of a deaf or hard-of-hearing child.

Of the 36 total participants, 29 self-identified as deaf, with varying degrees of hearing loss and a range of communication preferences, including LSEC, spoken Spanish, and written Spanish. The remaining 7 were hearing participants, including parents of deaf children and staff at a deaf school, whose perspectives were included to capture a broader understanding of community context and caregiving experiences. Coded identifiers were assigned to each participant to ensure confidentiality and were used consistently throughout transcription and analysis. Brief demographic details are provided in Table 1.

## 3. Data Collection and Materials

Interviews and focus groups (Morgan, 1997) were conducted from September to December 2024 and lasted approximately one hour. The language used during these sessions aligned with the preference of the participant; for those using LSEC, they interviewed with a deaf, fluent LSEC signer. For those who preferred spoken Spanish, they interviewed with a hearing trilingual interpreter. Interview locations were selected to support participant comfort, privacy, and communication access, including community spaces and other mutually agreed-upon settings. With participant permission, interviews were audio- or video-recorded using encrypted platforms. In total, data were collected across eight individual interviews and five focus groups; focus groups ranged in size from 3 to 9 participants, with an average of 5 members per group.

Semi-structured interviews (Patton, 2015) followed a pre-prepared interview guide designed to ensure consistency across interviews while allowing flexibility for participants to elaborate on topics most salient to their experiences. Interviews opened with introductory questions inviting participants to describe themselves and their experiences as deaf individuals in Ecuador, establishing both context and rapport. Participants were then asked to reflect on what quality of life means to them personally, followed by open-ended questions exploring major challenges encountered in daily life. Additional questions addressed how participants access information, communicate, and maintain connections with the deaf community. The list of questions can be found in Appendix A.

A subset of questions was tailored to specific subgroups, including deaf women, a deafblind participant and deaf individuals in rural areas, to examine intersectional experiences and structural barriers. Across all interviews, the guide addressed domains including quality of life, access to services, communication, health, education, employment, safety, and community inclusion, with prompts adapted to reflect individual participants’ priorities and experiences. Interviews were conversational in nature, allowing topics to emerge organically while remaining aligned with the study objectives.

### Data Analysis

All interviews were transcribed and analyzed in English using thematic analysis (Braun & Clarke, 2019), following six phases. Analysis followed an iterative and inductive analytic approach, in which codes were developed from recurring concepts, phrases, and meanings across transcripts and organized into broader themes representing shared experiences and priorities related to quality of life (Saldaña, 2011). The analysis focused on participants’ accounts of access to communication, healthcare, education, employment, safety, housing, information, and social inclusion, with attention to both patterns and variation across participants. For example, the following comments were grouped into a theme reflecting systemic educational barriers: inaccessible schooling, denial of sign language, unequal academic evaluation, and limited employment opportunities, whether due to educational tracks that restricted deaf individuals to certain jobs or employer discrimination that excluded them from broader opportunities. Themes were refined iteratively until they accurately and comprehensively represented participants’ perspectives.

Given the constructivist epistemological orientation of this study, data analysis followed reflexive thematic analysis (Braun & Clarke, 2019), in which analytic rigor is grounded in reflexivity, transparency, and collaborative interpretation rather than statistical inter-rater reliability. Accordingly, we did not calculate reliability coefficients, as consistency was established through iterative, consensus-based engagement with the data.

Four members of the research team independently familiarized themselves with and coded the transcript using the structured codebook. The team then met in debriefing sessions to compare coding decisions, discuss divergent interpretations, and refine code definitions through reflexive discussion and agreement. This process was repeated across several rounds of coding until no new substantive disagreements emerged.

Following coding, all team members collaboratively reviewed coded excerpts and grouped them into preliminary patterns of shared meaning, initially identifying four candidate themes. These groupings formed the basis for candidate themes, which were then developed and refined through an iterative, team-based, reflexive process. Themes were reviewed against the full dataset to ensure coherence, depth, and consistency across cases. Where overlaps, inconsistencies, or weakly supported themes were identified, they were merged or removed through collaborative discussion. Divergent and negative cases were explicitly examined and incorporated, and were analytically meaningful, either as nuance within existing themes or as distinct patterns. This iterative process continued across multiple rounds of review until the five final thematic structures were agreed upon by the research team. To enhance transparency, the codebook and representative coded excerpts are provided in Appendix B. Focus group and individual interview data were analyzed using the same overall analytic framework, but with attention to their distinct characteristics. Individual interviews were treated as accounts of personal experience, allowing for in-depth exploration of individual narratives. In contrast, focus group data were analyzed for both content and group dynamics, including points of agreement, disagreement, and the ways participants collectively constructed meaning. While all transcripts were coded using the same codebook to ensure consistency, segments from focus groups were additionally examined for interactional context. Findings from both data sources were then compared and integrated to identify convergent and divergent themes across individual and group-level data.

Given the multilingual and multimodal nature of this study, conducted across LSEC, spoken Spanish, and written Spanish, translation and transcription were treated as both ethical and analytic processes. Following (Brislin, 1970) back-translation method, professional sign language interpreters, bilingual research assistants, and deaf members of the research team collaborated to render interview content from LSEC and Spanish into English, attending carefully to the linguistic and cultural dimensions of participants’ responses. Deaf researchers played a central role in this process, interpreting meaning and flagging nuances that might otherwise be lost in translation. Where full equivalence across languages was not achievable, decisions were made collaboratively through team consensus. Member checking (Lincoln & Guba, 1985) was used to verify that translated content accurately reflected participants’ intended meanings. We acknowledge that some subtleties inherent to LSEC or Spanish may not be fully conveyed in the English translation.

Reflexivity was also incorporated during the analytic phase, distinct from the reflexive practices used during data collection. While our team’s shared deaf identities and community ties facilitated trust and communication access during data collection, these same positionalities required careful reflexive management during analysis. As deaf researchers analyzing accounts of structural barriers that many of us have personally encountered, we were aware of the risk of overidentifying with certain participants’ experiences, selectively weighing narratives that resonated with our own, or under-scrutinizing patterns that aligned with our prior expectations.

To address this, analytic reflexivity was maintained through several mechanisms. First, reflexive memos (Braun & Clarke, 2019) were written by each coder throughout the process, documenting interpretive decisions, moments of uncertainty, and instances where a coder’s positionality may have influenced a coding choice. Second, debriefing sessions were explicitly structured to include deliberate consideration of divergent or unexpected findings, ensuring that the analysis was not driven solely by convergence with anticipated themes. Third, the interdisciplinary composition of the team, spanning deaf education, linguistics, public health, and biomedical sciences, provided varied analytical perspectives that functioned as a form of internal peer scrutiny. Where team members with different disciplinary backgrounds interpreted the same segment differently, these differences were treated as analytically productive rather than resolved by deferring to the most senior researcher.

## 4. Results

Results are organized according to five interrelated themes that emerged from thematic analysis using an emic coding approach (Markee, 2013), in which recurring keywords and phrases across transcripts were identified and used to organize participants’ accounts. Together, these themes capture how structural barriers shaped the quality of life among deaf Ecuadorians. Consistent with the Structural Vulnerability Framework (Quesada et al., 2011), the themes reflect how political, economic, and institutional conditions, rather than individual limitations, produce patterned disadvantages across education, employment, healthcare, and daily life.

1. *Educational Exclusion and Inaccessibility* encompassed participants’ descriptions of barriers to accessing, remaining in, or succeeding within educational systems.
2. *Economic and Transportation Constraints* reflected accounts of financial hardship, unemployment or underemployment, transportation limitations, and the costs associated with education, healthcare, assistive devices, and basic services.
3. *Mistrust in Government and Institutional Support* emerged from narratives describing corruption, mismanagement of resources, lack of accountability, and repeated experiences of unmet needs within public institutions and deaf organizations.
4. *Excluded from Information and Voice* captured participants’ descriptions of communication barriers that limited access to information, healthcare, legal protection, and participation in decision-making.
5. *Lack of Family Support and Access to Resources* emerged from accounts of family-level neglect, adverse childhood communication experiences, control, or misunderstanding of deafness.

Across these themes, participants linked educational access, economic stability, institutional trust, information access, and family support to everyday well-being and autonomy.

### 4.1. Educational Exclusion and Inaccessibility

This theme captured participants’ experiences of limited, unequal, and often conditional access to education across primary, secondary, and postsecondary settings. Participants described education as a system that frequently excludes deaf learners, even when their academic performance is strong. For example, Participant 5 explained how educational systems privileged spoken and written language over academic achievement itself:

“Take my nephew, who has a cochlear implant. He never gets lower than 10 out of 10, but he doesn’t get his diploma. Because there’s another kid who knows how to speak and write ‘correctly.’ And that kid gets 10s and 9s, while my nephew, who gets pure 10s, doesn’t get his diploma.”

Others described being actively rejected by schools despite demonstrated capability, highlighting a pattern of institutional gatekeeping that undermines deaf students’ opportunities. Participant 9 expressed frustration with these exclusions, stating:

“They’re kicked out, they’re never accepted. I asked a teacher what could be done because a deaf person is capable of studying. That’s where I started to lose my patience…”

Access to higher education was perceived as largely unattainable, often contingent on substantial financial resources, further reinforcing structural inequities for disabled and deaf individuals in Ecuador. Participant 4 referenced broader societal narratives about disability and educational opportunity, stating, “There are movies where an autistic child becomes a great doctor in the United States or Europe. But in this country, to get an autistic child into a university, you’d need to be a multimillionaire.” This comparison reflected participants’ perceptions that meaningful educational advancement for disabled individuals was structurally inaccessible within Ecuador. Educational trajectories for deaf students were frequently narrowed toward manual labor or vocational skills rather than academic or administrative paths, limiting future employment opportunities. As Participant 36 described,

“I moved from [place] to [place] in search of better job opportunities, but I still struggle. My high school education allowed me to secure a cleaning job.”

Language practices within schools compounded these barriers. Participant 21 reported being forced to use oral speech while being prohibited from using sign language, the mode of learning most accessible to them:

“They forced me to learn how to speak orally and forbade me from using sign language to communicate and learn. Sign language is the best way for me to learn, but they wouldn’t let me.”

In contrast, schools that supported LSEC were described as critical for fostering independence and academic success. Participant 22 emphasized the importance of sign language-based education in shaping positive educational trajectories and autonomy for deaf students.

Consistent with the Structural Vulnerability Framework, these experiences reflect how institutional norms that privilege hearing communication systematically devalue sign language and position deaf students for lifelong disadvantage from an early age. Participants emphasized that even when students successfully navigated these barriers through informal support, the pathways remained precarious and insufficient, underscoring that educational exclusion is both structural and enduring.

### 4.2. Economic and Transportation Constraints

This theme reflects participants’ accounts of how financial strain and limited transportation shaped their access to essential services. Participants described how financial limitations, insufficient transportation infrastructure, and employment discrimination collectively constrain daily life and quality of life for deaf individuals in Ecuador. The costs of private healthcare, specialized schooling, interpreters, hearing aids, and other resources often exceeded what families could afford, leaving many unable to access essential services. Participant 4 described the cumulative burden of these expenses:

“Besides paying tuition, you have to pay for supplies, for one thing. And you’re required to pay for transportation. It’s almost 400 dollars a month.”

Participant 1 similarly described the burden of health costs in addition to navigating deafness when public systems proved inaccessible:

“I manage to maintain a somewhat normal life, but it greatly affects my finances. Often, when I get sick and need to visit the doctor, I rarely use the Social Security system because it is very difficult to get immediate appointments, so I pay for private doctors, clinics, and medicines.”

The cost of assistive devices further compounded financial strain. Participant 17 described the burden of maintaining hearing aids:

“We’ve gotten hearing aids for my deaf daughter, but when they need to be fixed, it’s way too expensive. We can’t afford it.”

Access to interpreters is similarly constrained by cost. Participant 12 noted:

“We could get an interpreter, but we have to pay for it, and we don’t have money. I know only one interpreter, but they’re usually busy.”

These financial constraints were compounded by transportation challenges, as educational, medical, and social resources are often centrally located, requiring multiple bus trips or expensive door-to-door transport, which participants also described as unsafe. Participant 1 commented on how insecurity further restricted mobility and autonomy:

“Due to insecurity, I cannot travel to distant places because of the risk of assaults on public or private transportation.”

Barriers to employment further illustrate the economic impact of these intersecting conditions. Deaf individuals frequently encountered workplace discrimination, lower wages, and limited job opportunities even when they had relevant skills or education. Participant 5 described how disabled workers were explicitly offered reduced compensation:

“We need equality! Because in various jobs, a disabled person is marginalized. And besides being marginalized, they don’t pay them a full salary. ‘Oh, you’re disabled? You can have half a normal salary.’”

Participant 5 also recounted how access to work experience depended not on qualifications but on personal connections:

“As soon as they saw his ID, they said no, he’s disabled. Only through a friend I know was he able to do his work study. If I hadn’t had that connection, he wouldn’t have gotten it.”

Access to training programs often depended on informal networks rather than formal pathways, reflecting systemic inequities in employment. Within the Structural Vulnerability Framework, the combination of limited financial resources, inaccessible transportation, and discriminatory labor practices illustrates how economic and structural conditions reinforce one another, leaving many deaf individuals reliant on informal work or family support and further constraining their opportunities for independence and social participation.

### 4.3. Mistrust in Government and Institutional Support

This theme captures participants’ widespread mistrust of government institutions, public services, and disability and deaf organizations. Participants consistently described political and economic instability as limiting access to essential services and resources, and linked corruption, misappropriation of funds, and bureaucratic inefficiency to the failure of aid, medical care, and educational support to reach deaf individuals. Participant 7 noted how access to services was shaped by influence and financial power rather than need:

“There are good centers, but because they are good, you can only enroll if you have pull and money.”

A particularly illustrative account came from Participant 3, who described repeated delays, astronomical costs and unnecessary procedures when seeking audiological services:

“It’s $80 for an exam. This one cost me $1080. When I went to the Ministry of Public Health, they made me do this test. Then they said it was no good and sent me to get another test. And without this test they would not give me the diagnosis. Then I went back with the new test and they told me they were going to wait for the government to send a different audiometry test. I had to wait a long time. I said, are you kidding me?”

Participant 4 similarly described structural neglect within public services, such as public hospitals and civic centers, which lacked hearing aids or failed to provide critical care due to insufficient supplies and administrative neglect:

“Government entities, public hospitals, health centers, civic centers, none have hearing aids for people. I lost my hearing because of an operation they should have done on my eardrum, but didn’t. There were no supplies; there was nothing like that. These issues cause people to become disabled, sometimes due to government negligence. I didn’t want to become deaf. I wanted the operation, but I didn’t have the resources to go to a private clinic.”

Participants also expressed frustration with the gap between government programs and meaningful community impact, perceiving that even well-intentioned initiatives rarely translated into tangible support. In some cases, deaf-serving organizations were described as prioritizing institutional reputation over community needs, reinforcing the perception that resources are distributed based on influence or personal connection rather than need. As Participant 15 spoke of diminished trust:

“Deaf-serving organizations don’t truly help me with my needs as a deaf person. They seem to care more about their reputation. Very few people actually help me with what I need, so I’m careful about who I open up to.”

These accounts reflect what the Structural Vulnerability Framework characterizes as the cumulative effects of structural abandonment: repeated institutional failures that produce not passivity, but informed skepticism grounded in lived experience. This mistrust further compounds vulnerability by discouraging reliance on the formal systems ostensibly designed to provide support.

### 4.4. Excluded from Information and Voice

This theme highlights how participants were systematically excluded from access to critical information and from meaningful participation in decisions affecting their lives. Participants described being cut off from information in both public and private contexts, shaping their ability to make decisions, access services, and participate fully in society. In healthcare settings, reliance on family members or peers to interpret often led to incomplete understanding, embarrassment, and mismanagement of health needs. Participant 12 shared how this dynamic shaped their experience of care:

“I go to the hospital with my mom to help with interpreting, but she doesn’t fill me in with everything. She takes over and talks with the doctor. I just sit back and wait for the conversation to be done. I am sometimes embarrassed to tell my health problems to my mom, so I just keep it to myself.”

This reliance on family interpreters was itself a structural problem rather than a solution. Participant 30 noted that formal interpreter support was rarely provided:

“Doctors won’t pay or find interpreters; we have to use family members to interpret for us.”

Other participants described similar breakdowns in communication that directly affected their understanding of treatment. Participants 30, 32, and 33 reported confusion about medications and medical instructions, emphasizing that health information was often delivered in ways they could not fully access or verify, leading to uncertainty and potential health risks:

Participant 30: “Doctors don’t take the time to explain health problems or medications.”

Participant 32: “My doctor told me that I need to do this and that but I don’t understand what I’m supposed to do.”

Participant 33: “My doctor told me to take this and that made my health worse. There is too much confusion and a communication barrier.”

Participants also described exclusion from public information and civic life, with news and organizational updates either absent, inaccessible, or inconsistently provided. This exclusion carried serious physical, social, and psychological consequences, particularly when it intersected with situations of violence and lack of legal protection. Participant 16 described the situation of a deaf woman who had experienced repeated abuse and was unable to access justice:

“I have a deaf woman friend that kept getting abused, molested, raped and taken advantage of. I am struggling with helping her because I can’t afford interpreters to help her make a report in court.”

The absence of qualified interpreters in legal settings prevented deaf individuals from reporting abuse or accessing protection, illustrating how informational exclusion is directly linked to exposure to harm. Consistent with the Structural Vulnerability Framework, these accounts demonstrate how communication access functions as a structural condition that mediates exposure to harm. Deaf people are not simply underserved; they are systematically denied the ability to participate in decisions that affect their own lives, reinforcing existing inequities and compounding vulnerability across multiple domains.

### 4.5. Lack of Family Support and Access to Resources

This theme reflects how limited family understanding of deafness, combined with scarce access to supportive resources, constrained participants’ independence, development, and wellbeing. Participants described how deaf individuals often face significant limitations in communication support from families and caregivers, affecting access to education, language development, and independent living.

A central barrier was families’ lack of knowledge about deafness and sign language. Participant 22 described how widespread misconceptions shaped the support deaf children received:

“Many families and professionals mistakenly believe that children will ‘grow out’ of their hearing loss or that they will naturally learn to speak. These misconceptions prevent deaf children from receiving the appropriate guidance and resources they need. This lack of awareness often leads to children being sent to mainstream hearing schools or special education programs meant for disabilities unrelated to deafness.”

Even when families recognized the need for sign language, access to instruction remained a significant barrier. Participant 6 described the limitations of available learning options:

“Where I am, mothers don’t know sign language. We don’t know, honestly we don’t know. They have online schools, as they say. There are classes online, but it’s difficult to learn that way.”

Many parents lacked knowledge of sign language or the financial resources to learn it, making informal or online learning inadequate substitutes for structured instruction. Participant 17 described this gap in access to language and support:

“I’ve met with other deaf people who use sign language, but I struggle with understanding them. I’d like to learn sign language; it would benefit my daughter, but I don’t know where to learn it. I feel bad for her because sometimes she would cry and get sad because her dad would yell at her for not understanding him. We have not gotten any resources or services from anyone about deafness. I don’t know anything about FENASEC.”

Even when deaf individuals had peers or relatives who could sign, understanding was often inconsistent, leaving them with few avenues for learning or support. Participant 27 stated the absence of support in direct terms:

“My family neglected me and doesn’t support me because I’m deaf.”

Beyond communication barriers, participants also described how families could restrict social participation and autonomy, sometimes confining or controlling deaf individuals’ movements due to fear, stigma, or misunderstanding. Participant 24 reported severe restrictions experienced by some deaf individuals in their communities:

“I know there are some deaf people that are chained up.”

Participant 19 recalled childhood punishment for using sign language:

“Hearing people hit me because I used sign language to communicate.”

Two participants described adults and older deaf individuals being isolated or prevented from attending community events (Participants 25 and 29). These experiences illustrate how exclusion operates across the life course, beginning in childhood and extending to adulthood.

Within the Structural Vulnerability Framework, these patterns of family-level neglect and control are understood not as individual failures but as outcomes of structural abandonment. In the absence of publicly available sign language education, family counseling, and community-based support, responsibility for deaf individuals’ care and communication is transferred onto households that are often unprepared and unsupported. The result is that institutional neglect intensifies dependence and constrains autonomy, particularly for those who lack access to sign language, community networks, or economic resources.

## 5. Discussion

This study provides a deaf-led, participant-centered account of how structural, institutional, and interpersonal barriers collectively shape the quality of life of deaf people in Ecuador. Across themes, participants’ accounts demonstrate that exclusion is not produced by any single barrier, but as the cumulative effect of inaccessible education, economic precarity, limited transportation, restricted access to information, weak institutional accountability, and pervasive barriers to communication access. Barriers were consistently interconnected, with communication exclusion operating as the underlying mechanism through which inequities were reproduced across domains of daily life.

A central contribution of this study is the demonstration that communication access functions as an organizing condition across all quality of life domains rather than as a discrete accommodation (Quesada et al., 2011). Participants’ narratives show that the absence of LSEC in education, healthcare, and public life does not simply create inconveniences; it actively restructures life trajectories by limiting educational attainment, narrowing employment possibilities, delaying or distorting health information, and restricting civic participation. In this sense, communication access operates as a structural determinant that mediates exposure to poverty, insecurity, and institutional harm (Groce et al., 2011; Quesada et al., 2011; West, 2022). These findings underscore that deaf people’s marginalization is not rooted in deafness itself, but in systemic failures to provide accessible support (Kuenburg et al., 2015; Lane, 1993; Meulder, 2015; Shakespeare, 2013; West, 2022).

From a structural vulnerability perspective (Quesada et al., 2011), educational exclusion operates as an early and enduring mechanism through which deaf people are positioned for lifelong disadvantage. Participants’ experiences of oralism, diploma denial, and inaccessible classrooms reflect institutional norms that privilege hearing communication and systematically devalue sign language (M. L. Hall et al., 2019; W. C. Hall, 2017; Meulder, 2015). Prior research has documented the linguistic and educational characteristics of the Ecuadorian deaf community (Eberle et al., 2012), and qualitative work has highlighted parental concerns regarding limited educational opportunities for deaf children (Huiracocha-Tutiven et al., 2017). The present study extends this literature by centering deaf individuals’ own accounts, demonstrating how educational inaccessibility functions as an early mechanism of structural vulnerability that shapes employment prospects and long-term quality of life.

Exclusion from information and voice emerged as a distinct axis of structural vulnerability, with direct consequences for health, safety, and legal protection (Bauman, 2004; Chavez, 2018; West, 2022). Recent research has documented communication barriers and dissatisfaction with healthcare access among deaf Ecuadorians (Izquierdo-Condoy et al., 2024), as well as the role of accessible information platforms in mitigating reproductive health inequities (Robles-Bykbaev et al., 2019). Participants in the present study described similar barriers, including reliance on family members for interpretation and lack of access to emergency information. Within the Structural Vulnerability Framework, this silencing represents a form of symbolic and practical violence whereby deaf people are systematically denied the ability to participate in decisions that affect their own lives.

Participants’ widespread mistrust of government and disability-serving institutions reflects what Quesada et al. (2011) describe as the cumulative effects of structural abandonment. Repeated experiences of corruption, unfulfilled promises, and misallocated aid have produced not disengagement, but informed skepticism grounded in lived experience (Farmer, 2004; Groce et al., 2011; West, 2022). This mistrust further compounds vulnerability by discouraging reliance on the formal support systems that were ostensibly designed to address community needs.

Prior research has emphasized the concerns and expectations of families raising deaf children in Ecuador (Huiracocha-Tutiven et al., 2017), yet participants’ accounts reveal how family dynamics are themselves shaped by the absence of accessible, system-level support. Without publicly available sign language education, counseling, respite services, or guidance for families, responsibility for care and communication falls onto households that are often unprepared and unsupported (Eberle et al., 2012; Groce et al., 2011; Huiracocha et al., 2015; Huiracocha-Tutiven et al., 2017; McCann, 2023). While some families responded protectively, others resorted to restricting movement, exerting control, or becoming neglectful. These outcomes, however, reflect structural abandonment rather than family-level failures. Within the Structural Vulnerability Framework, these patterns illustrate how institutional neglect intensifies dependence and constrains autonomy, particularly for deaf individuals who lack access to sign language, community networks, or economic resources.

Addressing these inequities requires structural interventions extending beyond awareness or accommodation, including sustained investment in accessible services, accountability in governance, and robust support systems for deaf individuals and their families.

## 6. Limitations

This study has several considerations for interpretation. The qualitative design and purposive sampling strategy were intentionally selected to enable in-depth exploration of participants’ lived experiences, prioritizing depth of understanding over breadth of representation. This approach complements quantitative components better suited to questions of generalizability and population-level patterns. Together, these methodological approaches serve different but equally valuable purposes: where quantitative work captures scope and prevalence, qualitative inquiry illuminates the meaning, context, and complexity behind those patterns.

Because the study relied on self-reported accounts of lived experience, participants’ narratives reflect their interpretations of events and institutions. These accounts are essential for understanding quality of life and structural vulnerability, but do not allow for independent verification of institutional practices or policy implementation. Accordingly, the findings reflect the specific experiences of participants within the context studied and are not intended to represent all deaf Ecuadorians. Participants were primarily recruited through deaf community networks and associations, which may have resulted in the underrepresentation of deaf individuals who are more socially isolated, have limited community ties, or are language-deprived. Although participants included individuals with intersecting identities such as deaf women, a deafblind participant, and deaf individuals living in rural areas, the sample size did not permit in-depth comparative analysis across these subgroups. Intersectional differences were identified descriptively but could not be examined systematically. Future research employing larger samples or targeted recruitment of specific subgroups would help to address this gap.

Although efforts were made to capture linguistic nuance, the translation process from (LSEC) and Spanish into English may have resulted in partial loss of meaning. Sign languages are visually embodied and culturally situated, and some expressions, affective elements, and contextual meanings may not be fully conveyed through written English translation. While translations were reviewed carefully by deaf members of the research team, this limitation is inherent in cross-linguistic qualitative research.

### Future Research

Future research should build on these findings by conducting longitudinal studies to examine how educational access, communication support, and institutional trust shape deaf individuals’ life trajectories over time. Following deaf participants across key transitions, such as schooling, entry into the workforce, and aging, would provide insight into how early structural barriers produce cumulative disadvantage.

Future studies should further examine intersectional experiences within the deaf population, including differences related to gender, sexuality, rural residence, deaf blindness, and socioeconomic status. Focused studies on these subgroups would help clarify how structural vulnerability operates differently across social positions and identify where targeted interventions may be most needed.

Additional research should incorporate the perspectives of family members, educators, healthcare providers, and policymakers, alongside evaluations of existing programs and resources, to better understand how institutional practices are shaped and sustained and where opportunities for structural intervention exist. Comparative research examining deaf communities across Latin American countries could further illuminate how national policy environments shape quality of life outcomes.

Finally, community-driven participatory research remains essential. Continued involvement of deaf researchers and deaf community organizations in study design, data interpretation, and dissemination will be critical for producing knowledge that is both ethically grounded and practically relevant. Future research should also examine how community-based initiatives interact with, supplement, or compensate for gaps in state-supported services, while remaining attentive to the risk of normalizing community burden as a substitute for institutional responsibility.

## 7. Conclusions

Our study shows that the challenges faced by deaf individuals in Ecuador are produced through interconnected structural conditions rather than isolated barriers. Drawing on the Structural Vulnerability Framework (Quesada et al., 2011), participants’ accounts illustrate how educational inaccessibility, economic and transportation constraints, exclusion from information, and institutional neglect interact to shape everyday life, systematically restricting access to education, healthcare, employment, and civic participation.

These findings demonstrate that these disadvantages are not inherent to deafness but rather emerge from institutional failures that normalize inaccessibility and shift responsibility onto individuals and families. The absence of provider-supported resources leaves families to navigate deafness without adequate guidance, often deepening deaf individuals’ dependence and social isolation. Mistrust in government institutions, meanwhile, reflects lived experiences of corruption, neglect, and unfulfilled promises rather than disengagement from civic life. Addressing these inequities requires system-level interventions, including accessible education, guaranteed interpretation across public services, family support programs, and meaningful inclusion of deaf Ecuadorians in policy development. Framing deafness through a structural vulnerability lens, in Ecuador and in comparable contexts, underscores that inclusion is not merely an accommodation issue but a matter of social justice.

## Figures and Tables

**Table 1:** Brief demographics of each participants.

Participant #	Hearing Status	Communication/Language Modalities	Role
1	Deaf	Written	Individual participant
2, 3, 4, 8, 17	Deaf	Spoken	Individual participant
5, 10	Hearing	Spoken	Family member
6, 9, 11	Hearing	Spoken	Parent
7, 22	Hearing	Spoken	Teacher
12, 13, 14, 15, 16, 18, 19, 20, 21, 23, 24, 25, 26, 27, 28, 29, 30, 31, 32, 33, 34, 35, 36	Deaf	Sign Language	Individual participant

## Data Availability

The datasets used and/or analyzed during the current study are available from the corresponding author on reasonable request.

## Note

1 Multidimensional poverty is a measure of poverty that extends beyond income to capture overlapping deprivations in health, education, and living standards. Individuals are classified as multidimensionally poor when they experience multiple basic deprivations across key indicators such as education, housing, nutrition, and access to services.

## Appendix A. Qualitative Interview Guide

**Deaf Community Research: Ecuador**

The interview guide was organized into a general module and subgroup-specific modules to reflect the comparative qualitative design across participant groups.

**General Questions (All Participants/Focus Groups)**

These questions were asked in all interviews and focus groups:

- Can you tell me a bit about yourself and your experiences as a deaf person in Ecuador?
- What does “quality of life” mean to you?
- What are some of the biggest challenges you face in your daily life as a deaf person in Ecuador? Deaf Women:
- How do you access information and stay connected to the deaf community?

**Additional Prompts (All Participants).**

These prompts were used flexibly to deepen discussion:

- Can you share a story or an experience that illustrates your perspective on quality of life?
- What are your hopes and dreams for the future of the deaf community in Ecuador?
- Is there anything else you would like to share about your experiences?

**Subgroup Module: Deaf Women.**

These questions were asked only in interviews with deaf women participants:

- How do your experiences as a deaf woman in Ecuador differ from those of deaf men?
- How do you feel about access to healthcare and reproductive health services as a deaf woman?
- How do your experiences as a deaf woman in Ecuador differ from those of hearing women?
- What aspects of life are most important to your overall well-being as a deaf woman?

**Subgroup Module: Deafblind Participants.**

These questions were asked only in interviews with deafblind participants:

- Can you describe the unique challenges you face in accessing information and communicating with others?
- What services or technologies have been most helpful in improving your quality of life?
- How can society be more inclusive of deafblind individuals?

**Subgroup Module: Deaf Participants in Rural Areas.**

These questions were asked only in interviews with participants living in rural areas:

- How does living in a rural area affect your access to education, employment, and healthcare services?
- What communication methods are most commonly used in your areas?
- Are there any resources or support systems specifically needed by deaf people living in rural areas?

**Notes on Guide Structure**

Some questions were adapted across groups to ensure both comparability across participant experiences and responsiveness to subgroup-specific contexts. Overlapping items were intentionally kept where they served to enable cross-group thematic analysis.

## Appendix B. Codebook

**Theme 1: Educational Exclusion and Inaccessibility.**

**Code**	**Definition**	**Inclusion Criteria**	**Example Quote**
Academic Achievement Undermined	Situations where deaf students perform well academically but are still excluded from certification or progression	High grades but denial of diploma, promotion, or recognition	“He gets perfect grades but doesn’t get his diploma because another child knows how to speak and write correctly.” (P5)
School Gatekeeping	Institutional exclusion from schools or programs despite eligibility or ability	Rejection from schools, denial of enrollment, barriers based on disability	“They’re kicked out, they’re never accepted.” (P9)
Oralism Enforcement	Forced reliance on oral speech and restriction of sign language use in education	Prohibition of sign language, forced speech training	“They forced me to learn how to speak orally and forbade me from using sign language.” (P21)

**Theme 2: Economic and Transportation Constraints**

**Code**	**Definition**	**Inclusion Criteria**	**Example Quote**
Financial Barrier to Services	Economic inability to access healthcare, education, or assistive devices	High cost of services, inability to afford care or education	“It’s almost 400 dollars a month for transportation and supplies.” (P4)
Employment Discrimination	Exclusion or unequal treatment in employment due to deafness/disability	Job denial, lower pay, workplace exclusion	“As soon as they saw his ID, they said no, he’s disabled.” (P5)
Mobility Restriction	Limited travel due to infrastructure, cost, or safety concerns	Unsafe transport, lack of access, inability to travel	“I cannot travel because of the risk of assaults on transportation.” (P1)

**Theme 3: Mistrust in Government and Institutional Support**

**Code**	**Definition**	**Inclusion Criteria**	**Example Quote**
Institutional Neglect	Failure of public systems to provide adequate services or resources	Lack of equipment, delays, missing services	“Public Hospitals… none have hearing aids for people.” (P4)
Corruption/Inequality in Access	Perceived unfair distribution of services based on money or connections	“Pull,” bribery, favoritism	“You can only enroll if you have pull and money.” (P7)
Organizational Distrust	Lack of trust in deaf-serving organizations or NGOs	Perceived self-interest, lack of transparency	“Deaf-serving organizations don’t truly help me with my needs as a deaf person. They seem to care more about their reputation.” (P15)

**Theme 4: Excluded from Information and Voice**

**Code**	**Definition**	**Inclusion Criteria**	**Example Quote**
Interpreter Barriers	Lack of access to qualified interpreters in critical settings	Absence of interpreters, unqualified interpreters, reliance on family	“I go to the hospital with my mom to help with interpreting, but she doesn’t fill me in with everything. She takes over and talks with the doctor. I just sit back and wait for the conversation to be done.” (P12)
Legal Communication Exclusion	Inability to access justice system due to communication barriers	Barriers to accessing court, inability to report abuse	“I can’t afford interpreters to help her make a report in court.” (P16)
Information Inaccessibility	Lack of access to public or health-related information	News, announcements, or instructions are not accessible	“Doctors don’t take the time to explain health problems or medications.” (P33)

**Theme 5: Lack of Family Support and Access to Resources**

**Code**	**Definition**	**Inclusion Criteria**	**Example Quote**
Communication Neglect in Family (Adverse Early Childhood Communication Experiences)	Lack of sign language or communication support within family	No shared language, inability to communicate	“My family neglected me and doesn’t support me because I’m deaf.” (P27)
Misconceptions About Deafness	Beliefs that deafness will resolve or does not require intervention	Expectation child will “learn to speak naturally”	“They assumed I would grow out of it.” (P22)
Family Control and Restriction	Limiting autonomy, mobility, or social participation	Confinement, punishment, restriction of movement	“I know some deaf people that are chained up.” (P24)

This codebook reflects a reflexive thematic analysis approach (Braun & Clarke, 2019), in which codes were developed iteratively and refined through team-based discussion. Codes represent interpretive patterns rather than fixed or exhaustive categories.
